# Seed Density Significantly Affects Species Richness and Composition in Experimental Plant Communities

**DOI:** 10.1371/journal.pone.0046704

**Published:** 2012-10-15

**Authors:** Zuzana Münzbergová

**Affiliations:** 1 Department of Botany, Faculty of Science, Charles University, Praha 2, Czech Republic; 2 Institute of Botany, Academy of Sciences of the Czech Republic, Průhonice, Czech Republic; Stockholm University, Sweden

## Abstract

Studies on the importance of seed arrival for community richness and composition have not considered the number of seeds arriving and its effect on species richness and composition of natural communities is thus unknown. A series of experimental dry grassland communities were established. All communities were composed of the same 44 species in exactly the same proportions on two substrates using three different seed densities.

The results showed that seed density had an effect on species richness only at the beginning of the experiment. In contrast, the effects on species composition persisted across the entire study period. The results do not support the prediction that due to higher competition for light in nutrient-rich soil, species richness will be the highest in the treatment with the lowest seed density. However, the prevalence of small plants in the lowest seed density supported the expectation that low seed density guarantees low competition under high soil nutrients. In the nutrient-poor soil, species richness was the highest at the medium seed density, indicating that species richness reflects the balance between competition and limitations caused by the availability of propagules or their ability to establish themselves. This medium seed density treatment also contained the smallest plants.

The results demonstrate that future seed addition experiments need to consider the amount of seed added so that it reflects the amount of seed that is naturally found in the field. Differences in seed density, mimicking different intensity of the seed rain may also explain differences in the composition of natural communities that cannot be attributed to habitat conditions. The results also have important implications for studies regarding the consequences of habitat fragmentation suggesting that increasing fragmentation may change species compositions not only due to different dispersal abilities but also due to differential response of plants to overall seed density.

## Introduction

Dispersal is considered one of the key processes affecting the richness and composition of natural communities, e.g. [1], [2], [3], [4]. Following MacArthur & Wilson [5], plant communities are predicted to be more diverse in areas with higher regional richness, which guarantees higher immigration rates of new species into the community [6]. The rate of immigration into a community is one of the key parameters in neutral models of community [7] and can be described by the composition of the arriving seeds, the time of seed arrival and the density of the arriving seeds, e.g. [8], [9], [10], [3].

The importance of seed arrival is usually studied by seed addition experiments in which changes in community and composition are tracked following the addition of seeds of different species, e.g. [11], [12], [13], [14], [15], reviewed by Myers & Harms [3]. The results of these studies generally show that seed arrival increases local species richness in a wide range of communities, and the increase in species richness is more pronounced in more disturbed communities [3].

In their review, Myers & Harms [3] identified two major limitations of seed addition studies. The first limitation is the lack of studies explicitly separating the relative roles of dispersal in limiting vs. maintaining diversity (but see Vandvik & Goldberg [16], [17]). The second is that there is little information available on the intensity of the natural seed rain, so the number of seeds added to the communities probably far exceeds the number that arrives naturally.

The aim of this study was to explore the effects of seed rain intensity on the richness and composition of simulated dry grassland communities by manipulating density of seeds used to set up experimental plant communities [18], [11], [19]. Two opposing predictions about the relationship between seed rain intensity and the resulting species richness are possible. First, it can be predicted that species richness will increase with higher seed rain intensity because this will increase the probability of establishment of each of these species or the intensity of positive interactions between species. Conversely, higher seed rain intensity may lead to a higher degree of competition between plants in the community. Higher competition is known to have a negative effect on species richness, and communities with higher seed rain intensities may consequently have lower species richness, e.g. [20]. These opposing predictions indicate that current seed addition experiments may either overestimate or underestimate the degree to which species richness is limited.

Each of the two opposing predictions can be expected to hold true for species with different competitive ability as suggested by the competition–colonization trade-off, e.g. [21], [22] and will also depend on productivity of the habitat as different productivity will lead to different levels of competitive asymmetry [23], [24]. Specifically, greater competition for light, which is typically asymmetric, would be expected in more productive habitats; higher competition for nutrients, which is typically symmetric, would be expected in less productive habitats, cf. [23]. Thus, it can be predicted that increased species richness with increased seed rain intensity is more likely to occur in communities with low productivity. Increased species richness with decreased seed rain intensity is more likely to occur in communities with high productivity [25], [26]. A similar pattern of changing limitation with habitat productivity was observed by Foster et al. [27] and was used to support the shifting limitations hypothesis [28], [29].

It has been repeatedly suggested that aboveground biomass produced by a community is related to strength of competition within the community, e.g. [30], [31]. This is because plants at sites with more biomass more strongly interact [32]. Others have argued that competition intensity should be independent on aboveground biomass, as competition reflects the ratio between resource demand and supply [33], [34]. This controversy reflects the fact that the effect of aboveground biomass will depend on resource availability in the environment. Under exactly the same environmental conditions, relationship between competitive intensity and aboveground biomass production should thus be expected. Thanks to this, aboveground biomass has been shown to be a useful predictor of species diversity in natural communities, e.g. [35]. Aboveground biomass is likely to depend on the type of substrate and will likely also change under different seed rain intensities. Thus, I predict that changes in aboveground biomass could be one of the factors responsible for changes in species richness in experimental communities and its effect will change between the two substrates. Based on previous studies on the relationship between productivity (often measured as aboveground biomass) and species richness, it is hard to predict the exact shape of the relationship as all decreasing, increasing as well as unimodal relationships have been previously reported, e.g. [36], [37], [38], [39], [40], [41]. In any case, if biomass was the main driver of changes in species composition, it can be predicted that the effect of density on species richness will become non significant after including aboveground biomass as a covariate into the models.

Changes in intensity of competition within a community will likely also changes species composition of the given community, e.g. [42], [43], [44]. The differences in the species composition of communities with different seed densities are expected to be non-random such that more competitive species are more successful at higher seed densities [33], [45]. Goldberg & Landa [46] and Rajaniemi et al. [47] predicted that tall species with high individual biomass and species that are most abundant in the field will be the strongest competitors and perform best at high initial seed densities. However, they did not confirm this prediction in an experiment examining annual plant communities. Nonetheless, other studies have predicted and found species abundance to be based on species traits, e.g., [48], [49]. On this basis, it can be predicted that species success under different seed rain intensities will depend on the species' biological traits. Specifically, it can be predicted that species which are more competitive (are taller and form more aboveground biomass) will be more successful in higher density where competition is intense, while less competitive species will prevail in low density treatments.

In this study, different seed rain intensities were simulated by varying the initial sowing densities used to establish in a perennial grassland community. The experiment was designed to answer the following questions: (i) What is the effect of seed rain intensity on aboveground biomass, species richness and species composition in experimental communities? (ii) How does the effect of seed rain intensity depend on the nutrient status of the soil? (iii) Can differences in species richness be explained by differences in aboveground biomass? (iv) Can we use species' traits to predict species' responses to seed rain intensity? This experiment studies the importance of different seed density for community diversity and composition. It is mimicking different seed rain intensity at the stage of establishment of new plant community on a disturbed habitat. The conclusions are, however, applicable also for studies dealing with seed rain into established plant communities under the assumption that the established community is mainly depleting resources and thus changing carrying capacity of the system.

## Methods

No specific permits were required for entering localities used for collecting material for the experiment as they were not privately-owned or protected in any way. The study did not involve endangered or protected species.

### Study systems and selected species

The simulated systems are calcareous dry grasslands in northern Bohemia, Czech Republic, Europe. Grasslands in this region form distinct localities mainly surrounded by agricultural fields. Many fields are currently abandoned and are undergoing succession towards dry grasslands [50], [51]. In previous studies of this system [52], [53], we showed that the distribution of many species is limited by dispersal and that the composition of the local communities depends on habitat isolation and size.

For the study, we used 44 perennial herb species commonly occurring species in the studied localities for which a sufficient amount of seeds were available. Thus, species that are rare or only produce small numbers of seeds were excluded (see Table S1 in Supporting Information). Seeds of most of the species were collected in the field. Seeds of a few species (5%) were obtained from a commercial seed producer in the region (Planta Naturalis Company). All seeds were stored in paper bags under standard room conditions (∼20°C) from the time of collection until sowing in the spring. Six species (*Anthericum ramosum*, *Aster amellus*, *Cirsium acaule*, *Coronila varia*, *Dianthus carthusianorum* and *Primula veris*) never germinated, and thus, only 38 species appeared in at least one pot.

To interpret the results, species were grouped into three size categories based on their biomass production. There were independent data on the maximum production of biomass per year (g of dry mass) for twenty-six of the species, based on Tremlová & Münzbergová [53]. The first category contained small plants usually up to 30 cm in height with little clonal growth and yearly aboveground biomass production up to 4 g of dry mass; the second category contained plants usually between 30 and 60 cm high with medium clonal growth and biomass production up to 17 g; the third category contained plants usually above 60 cm tall, often with extensive clonal growth forming large tussocks, and biomass production of more than 17 g. The species for which biomass data were not available were classified based on my knowledge of the species and their similarities to other species with known biomass production. Categories were also created based on plant height and intensity of clonal growth. The results according to classification based on plant height and intensity of clonal growth were similar but not as strong as those based on plant size; thus, only the results based on plant size are presented.

### Set up of the experiment

The experiment was conducted in an experimental garden at the Institute of Botany, Academy of Sciences of the Czech Republic, Průhonice (50°0′7.11″N, 14°33′20.66″E, 350 m asl.). The conditions in the garden were similar to those in the area from which the seeds were obtained (the region located at approximately 50°31′44.6″N, 14°15′12.6″E, 300 m asl., about 60 km from the garden).

A series of experimental dry grassland communities was established. All communities were composed of the same 44 dry grassland species and were established using three different seed densities that simulated different seed rain intensities in pots starting with bare soil (low, medium and high sowing density). All species were present in exactly the same proportions. Furthermore, the communities were established on two different substrates (nutrient-poor, nutrient-rich) which differed in nutrient availability (Table S2).

The medium seed rain intensity was based on the natural seed production of the constituent species. The field seed production was estimated in three populations of each of the species in the studied region. For each population, the number of developed seeds per plant was recorded for 20 randomly selected individuals. This number was multiplied by the number of flowering shoots per 1 m^2^. This was counted in five selected quadrats within a population of the species at the three localities. The quadrats were located in places with the highest densities of the focal species to capture the maximum density that the plants can achieve in the field. The low seed rain intensity represented 25% and the high seed rain intensity represented 400% of the medium seed rain intensity, respectively. The low and high seed rain intensity values were selected to cover as wide a range of seed rain intensities as possible while maintaining values that could theoretically occur in the field. Only three seed rain intensity levels were used to achieve a sufficient number of replicates for each level and to keep the materials required to set up the experiment manageable. Between 20 and 436 seeds per plant were sown into the pots in the medium seed rain intensity, depending on the species. In total, 1997 seeds were added to the medium seed rain intensity treatment.

The two substrates used in this study represent two habitat types occurring in the source region, i.e., the nutrient-poor localities of dry grasslands (soil taken directly from these localities) and the nutrient-rich sites that are found where dry grasslands were turned into arable fields and extensively fertilized before being abandoned (the soil taken from the experimental garden was similar to the soil in the abandoned fields, unpubl. data). A comparison of soil properties 1 year into the experiment is presented in Table S2. The plants were watered regularly (once per week) and also received natural rainfall. Thus, the plants were able to experience some water stress. I assumed that the level of water stress was similar to natural conditions at the localities. At the natural localities, plants received only rainfall. However, the soil at natural localities is much less prone to desiccation compared to soil in the relatively small, freely standing pots. However, no direct measurements are available to support this assumption.

Each experimental community was established in a circular pot 50 cm in diameter and 34 cm deep with 10 replicates of each treatment combination (i.e., 60 pots in total). The seeds were sown into the pots in the beginning of May 2007. The seeds were sown on the soil surface and then gently pressed into the soil to ensure that they were not blown away. All seeds were added simultaneously into the pots and thus possible priority effects are only due to differences in germination speed between species and not due to different sowing times. The pots were regularly inspected and all non-target species were weeded as soon as they could be reliably distinguished from the target species.

To determine the composition of the community, all aboveground biomass above 3 cm in height was harvested twice during the field season, in mid July and at the end of September, which corresponded to the first and second peaks of biomass production and mimicked the traditional management of grassland localities in the area. All harvested biomass was sorted into species, dried to a constant weight and weighed.

### Data analysis

All analyses are based on data from 3 years (2007–2009) of 2 harvests per year (July, September), resulting in 6 recordings referred to as time 1–time 6.

Repeated measures ANOVA was used to separately test the effects of seed rain intensity, substrate, time and their interactions on total aboveground biomass and species richness (number of species) in the experimental communities. The effects of seed rain intensity and substrate were also tested at each time interval for all dependent variables. The data on total aboveground biomass were square-root transformed to fit the assumptions of normality and homogeneity of variance. A transformation of species richness was not necessary.

It is possible that the effect of seed rain intensity on species richness could be mediated by total aboveground biomass. The above tests, which used species richness as the dependent variable, were thus repeated using aboveground biomass in the concurrent time period as a covariate. In this way, the possibility that aboveground biomass during a particular time period determined the species richness was explored. All analyses were conducted using S Plus [54].

Data on species composition were analyzed using Canonical Correspondence Analysis (CCA) in Canoco [55]. First, the effects of seed rain intensity, substrate and their interactions (used as independent variables) on species composition were analyzed with all time periods grouped together. Analyses were then conducted for each time period separately. The dependent variables in the CCA analyses were the biomass of each species or the presence/absence of each species in the experimental community. Because the results based on species biomass and species presence/absence were largely similar, only the results based on species presence/absence data are shown. I present the data on presence/absence rather than on species biomass because the presence/absence data more clearly show the differences in species composition and not simply in the proportions among species.

To analyze the effect of species traits on species response to seed rain intensity, I calculated species response to seed rain intensity as follows:
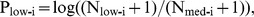
where P_low-i_ is a measure of prevalence of species i in low seed rain intensity compared to medium seed rain intensity, N_low-i_ is the number of pots occupied by species i in the low seed rain intensity treatment and N_med-i_ is the number of pots occupied by species i in the medium seed rain intensity treatment. Similarly, I calculated P_high-i_ by comparing occupancy in high and medium seed rain intensity pots. The P_low_ and P_high_ values were separately calculated for each time period and substrate as well as for all time periods and both substrates together. Because of strong differences in the results between the two substrates, only the results for each separate substrate are shown. The effects of plant size (defined above) and seed production per 1 m^2^ (corresponding to seed rain intensity in the medium seed rain intensity treatment) on P_low_ and P_high_ values were tested using ANOVA and linear regression, respectively. P_low_ and P_high_ values fitted the assumptions of normality and homogeneity of variance. I also used a similar index to compare plant abundance and not frequency. The results were very similar to the results based on species frequency and are thus not shown further.

## Results

**Figure 1:**
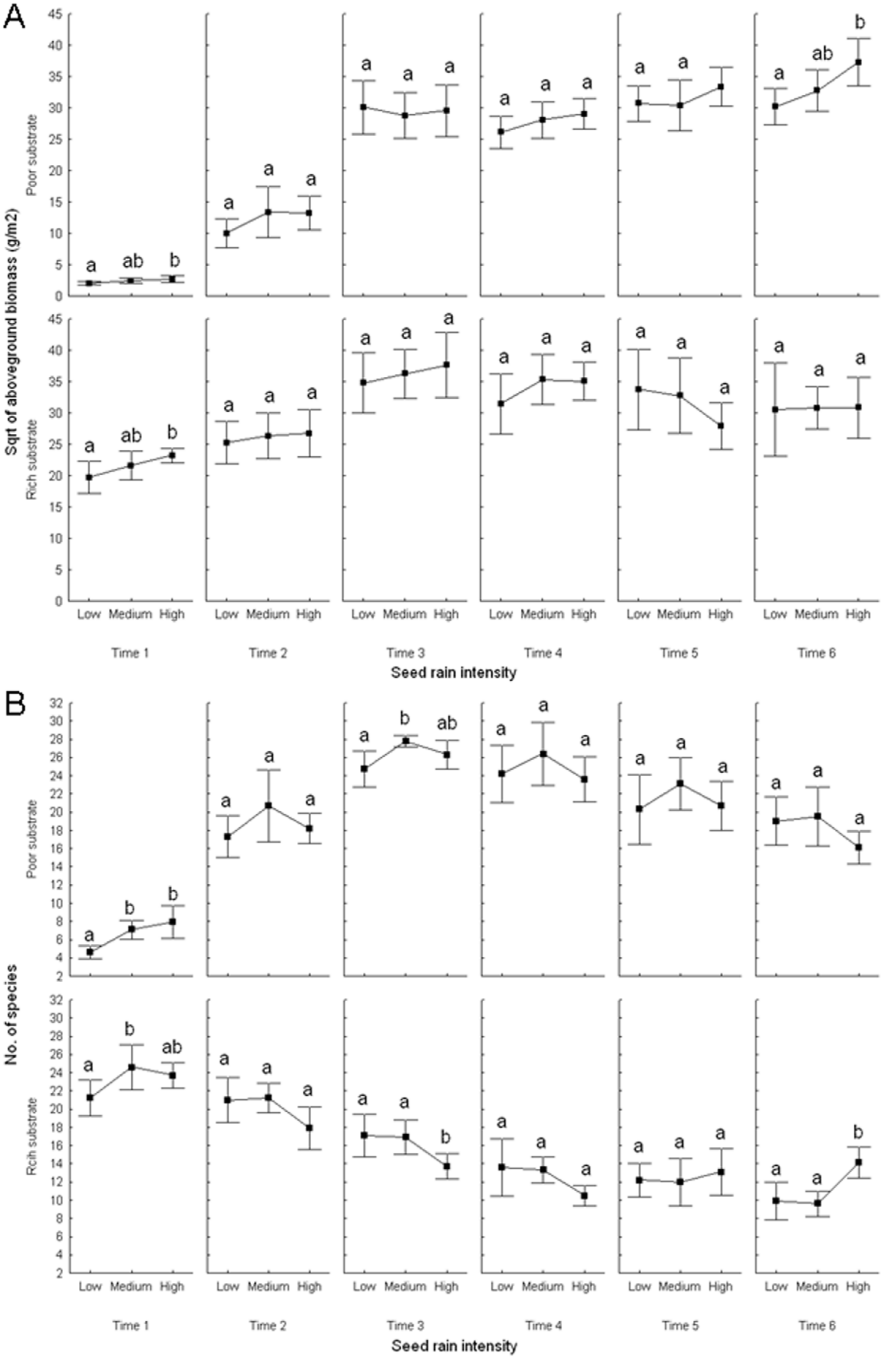
Effect of seed rain intensity, substrate and time on aboveground biomass and species richness. Effect of seed rain intensity, substrate and time on A) aboveground biomass (square root transformation) and B) species richness. The graphs show mean with 95% confidence intervals. Low, medium and high indicates low, medium and high seed rain intensity. Different letters indicate significant differences between given seed rain intensity treatments within substrates and times.

Seed rain intensity had no significant effect on total aboveground biomass (Table 1A; 2A). The effect of seed rain intensity on total aboveground biomass was not significant even in interaction with time and substrate (Table 1A). When analyzed separately for each time period, the effect of seed rain intensity on total aboveground biomass was significant only in the 1^st^ time period (with the highest biomass in the high seed rain intensity treatment). This suggests that biomass in the lowest seed rain intensity treatment reached the same level as in the highest seed rain intensity treatment in the first field season (Table 2A; Fig. 1A). Unlike seed rain intensity, substrate had a significant effect on total aboveground biomass through the 4^th^ time period (Table 2A; Fig. 1A). The amount of aboveground biomass was lower in the nutrient-poor substrate compared to the nutrient-rich substrate.

**Table 1:** Effect of seed rain intensity (seed rain), substrate and time on total aboveground biomass (A) and species richness (B) assessed using ANOVA and on species composition based on presence/absence of each species (C) assessed using Canonical Correspondence Analysis (CCA).

Dependent variable		A) Biomass		B) Species richness		C) Composition	
Type of analysis		ANOVA		ANOVA		CCA	
	df	var.	p	var.	p	var.	p
Seed rain	2	<0.01	0.117	*0.01*	*0.059*	**0.03**	**0.002**
Substrate	1	**0.11**	**<0.001**	**0.07**	**<0.001**	**0.07**	**0.002**
Time	5	**0.37**	**<0.001**	*0.01*	*0.06*	**0.1**	**0.002**
Seed rain×substrate	2	<0.01	0.51	0	0.337	**0.01**	**0.02**
Seed rain×time	10	<0.01	0.97	0	0.501	**0.02**	**0.006**
Substrate×time	5	**0.12**	**<0.001**	**0.3**	**<0.001**	**0.09**	**0.002**
Seed rain×substrate×time	10	<0.01	0.13	**0.01**	**0.048**	**0.06**	**0.002**

Var. indicates proportion of variance explained by the given independent variable from the total variation in the data (R^2^ values in case of ANOVA). Significant values p≤0.05 are in bold, marginally significant values p≤0.1 are in italics, n.s. indicates p>0.1. Df Error = 216.

**Table 2:** Effect of seed rain intensity (seed rain) and substrate on total aboveground biomass (A) and species richness (B) assessed using ANOVA and on species composition based on presence/absence of each species (C) assessed using Canonical Correspondence Analysis (CCA).

Dependent variable	Type of analysis	Independent variable		Time 1	Time 2	Time 3	Time 4	Time 5	Time 6
A) Biomass	ANOVA	Seed rain	var.	**0.01**	0.02	0.01	0.07	0.01	0.05
			p	**0.011**	0.208	0.797	0.064	0.72	0.19
		Substrate	var.	**0.95**	**0.69**	**0.25**	**0.3**	<0.001	0.04
			p	**<0.001**	**<0.001**	**<0.001**	**<0.001**	0.98	0.11
		Seed rain×substrate	var.	0.003	0.003	0.01	0.005	*0.092*	0.05
			p	0.126	0.726	0.655	0.812	*0.07*	0.24
B) Species richness	ANOVA	Seed rain	var.	**0.025**	**0.104**	**0.028**	*0.026*	0.01	0.00
			p	**<0.001**	**0.038**	**0.011**	*0.056*	0.58	0.78
		Substrate	var.	**0.909**	0.03	**0.789**	**0.733**	**0.579**	**0.505**
			p	**<0.001**	0.158	**<0.001**	**<0.001**	**0.00**	**<0.001**
		Seed rain×substrate	var.	0.002	0.05	**0.032**	0.01	0.02	**0.13**
			p	0.517	0.173	**0.006**	0.463	0.31	**<0.001**
C) Species composition	CCA	Seed rain	var.	**0.03**	**0.06**	**0.07**	**0.06**	**0.07**	**0.07**
			p	**0.1**	**0.004**	**0.004**	**0.01**	**0.002**	**0.002**
		Substrate	var.	**0.27**	**0.15**	**0.16**	**0.16**	**0.1**	**0.06**
			p	**0.002**	**0.002**	**0.002**	**0.002**	**0.002**	**0.002**
		Seed rain×substrate	var.	**0.08**	**0.05**	**0.05**	**0.05**	0.03	**0.09**
				**0.002**	**0.002**	**0.002**	**0.002**	0.25	**0.002**

The tests are done for each time period separately. Var. indicates proportion of variance explained by the given independent variable from the total variation in the data (R^2^ values in case of ANOVA, A and B). Significant values p≤0.05 are in bold, marginally significant values p≤0.1 are in italics, n.s. indicates p>0.1. Df Error = 54.

Aboveground biomass was also affected by the interaction between substrate and time (Tables 1A; 2A). On the poor substrate, biomass increased steadily over time. In contrast, biomass reached its maximum by time 3 on the rich substrate. Thereafter, biomass remained stable in the low seed rain intensity treatment and steadily declined in the medium and high seed rain intensity treatments. As a result, biomass was comparable in all treatments at time 6 (Figs. 1A and S1A).

Overall, seed rain intensity showed no significant effect on species richness (Table 1B). The effect of seed rain intensity, however, significantly interacted with time and substrate (Table 1B). The effect of seed rain intensity on species richness was significant from the 1^st^ to the 3^rd^ time periods, marginally significant in the 4^th^ time period and non-significant in subsequent time periods (Table 2B; Figs. 1B and S1B). The number of species in the high as well as low seed rain intensity treatments was lower than in the medium seed rain intensity treatment in all time periods.

The effect of seed rain intensity on species richness depended on the substrate, and this dependency changed over time (Tables 1B; 2B; Fig. S1B). In the nutrient-poor treatment, the number of species was the lowest in the 1^st^ time period and steadily increased until the 3^rd^ time period after which it decreased again (Figs. 1B and S1B). The decline was the least extreme in the low seed rain intensity and the sharpest in the high seed rain intensity treatments (Figs. 1B and S1B).

In the nutrient-poor substrate, species richness in the low seed rain intensity was significantly lower than in the medium seed rain intensity only in time periods 1 and 3. However, species richness in the high seed rain intensity treatment was never significantly different from the medium seed rain intensity treatment in the nutrient-poor substrate (Fig. 1B).

In the nutrient-rich substrate, species richness was the highest in time period 1 and then slowly decreased over subsequent time periods. The decline was the sharpest in the medium seed rain intensity. Species richness even started increasing again in the highest seed rain intensity treatment by time 6 (Figs. 1B and S1B). In the nutrient-rich substrate, species richness was significantly different between low, medium and high seed rain intensity only in some time periods (Fig. 1B).

When including the effect of aboveground biomass as a covariate, the effects of seed rain intensity on species richness remained almost unchanged. This suggests that the effect of seed rain intensity on species richness was independent of the effect of aboveground biomass (Table S3). The only exception was that the significant effect of seed rain intensity in time 1 was lost due to strong differences in biomass between the different seed rain intensity treatments in time 1 (Table S3).

Seed rain intensity had a significant effect on species composition in the experimental communities and strength of this effect increased over time (Tables 1C; 2C; Figs. S2 and S3). Its effect also significantly interacted with time and substrate (Table 1C). The significant effect of seed rain intensity on species composition was also visible in data from each separate time period, with the strongest effects in time 3, 5 and 6 (Table 2C).

**Figure 2:**
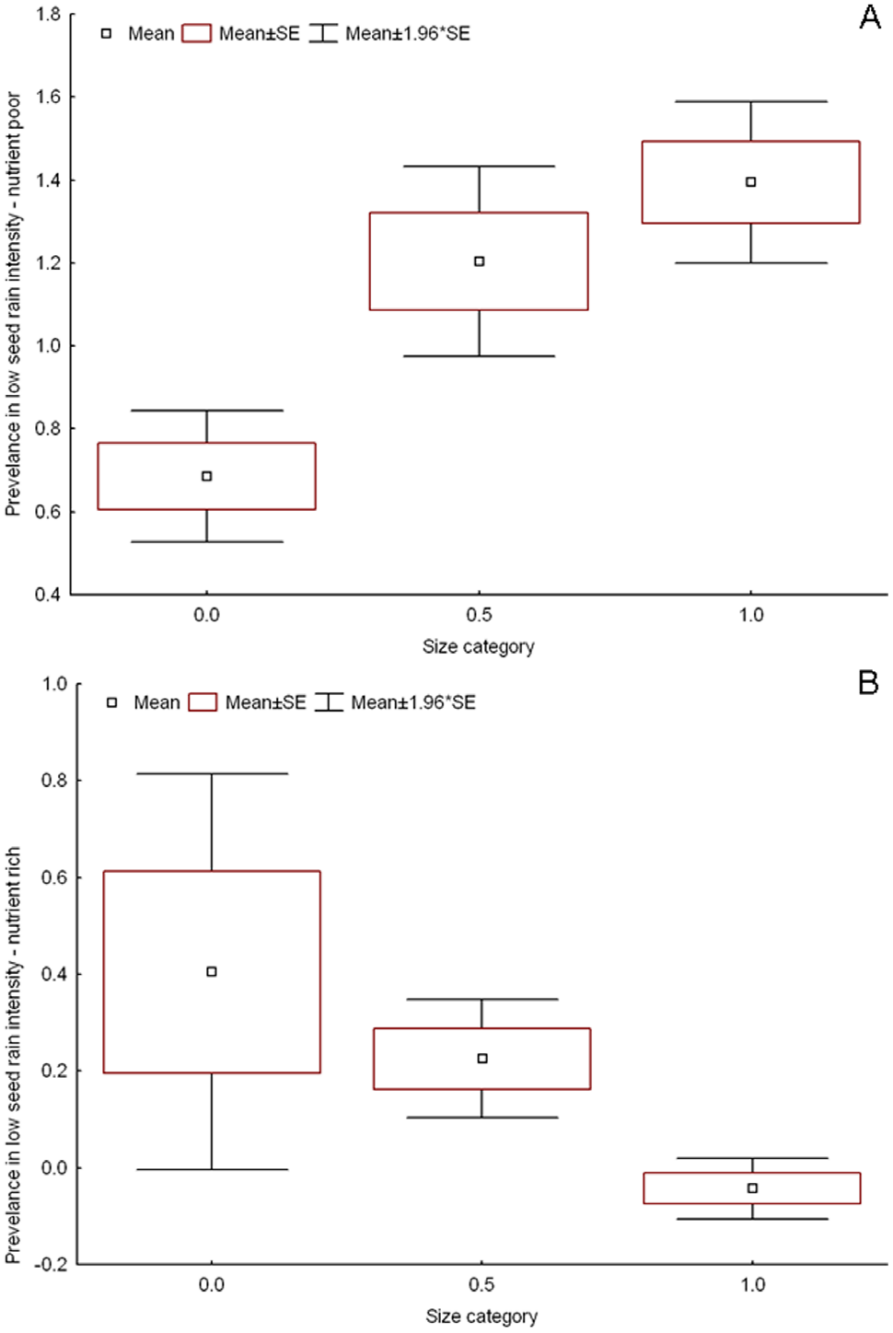
Effect of plant size on prevalence in low seed rain intensity in two substrates. Effect of plant size (3 size categories from small to large, data based on Tremlová & Münzbergová [53]) on species response to seed rain intensity estimated by comparing species presence in pots with low and medium and high and medium seed rain intensity expressed as prevalence values P_low_ and P_high_ (log of the frequency in low/high relative to frequency in medium seed rain intensity treatment) in nutrient-poor A) and nutrient-rich B) substrate. The graph is based on data combined over all time periods. Positive value indicates that species of the given size category are more common in the given seed rain intensity treatment compared to medium seed rain intensity.

Plant response to seed rain intensity could be explained by plant size, and this response strongly differed between substrates (Table 3). In the data for all time periods, small plants profited from low seed rain intensity in nutrient-rich substrate (Fig. 2), but large plants profited from low seed rain intensity in nutrient-poor substrate (Fig. 2). Large plants also profited from high seed rain intensity in nutrient-poor substrate. This indicates that in the nutrient-poor substrate, smaller plants are restricted to medium seed rain intensity. In contrast, in rich substrate, plants that prevailed in high seed rain intensity did not differ in size from plants in medium seed rain intensity. The same pattern was also visible in the 3^rd^ time period. Some of these relationships were also significant in other time periods (Table 3). In contrast to plant size, seed production per 1 m^2^ had no significant effect on species response to seed rain intensity (p>0.05 in all cases). This indicates that the initial seed number used to initiate the experiment for each species did not affect the outcomes.

**Table 3:** Effect of plant size (3 size categories from small to large, data based on Tremlová & Münzbergová [53]) on species response to seed rain intensity estimated by comparing species presence in pots with low and medium and high and medium seed rain intensity expressed as P_low_ and P_high_ values (log of the frequency in low/high relative to frequency in medium seed rain intensity treatment).

	Time	All			1			2			3			4			5			6		
Subs.	Seed rain	R^2^	p	Dir.	R^2^	p	Dir.	R^2^	p	Dir.	R^2^	p	Dir.	R^2^	p	Dir.	R^2^	p	Dir.	R^2^	p	Dir.
Poor	Low	**0.44**	**0.001**	**+**	0.01	0.674		**0.34**	**0.006**	**+**	*0.18*	*0.058*	*+*	0.10	0.152		0.07	0.244		**0.33**	**0.006**	+
	High	**0.24**	**0.024**	**+**	**0.19**	**0.048**	**+**	0.00	0.794		**0.30**	**0.011**	**+**	0.04	0.403		<0.01	0.996		0.03	0.474	
Rich	Low	**0.41**	**0.002**	**−**	0.02	0.561		**0.36**	**0.004**	**−**	**0.34**	**0.006**	**−**	0.04	0.385		**0.20**	**0.041**	**−**	0.14	0.097	
	High	<0.01	0.858		0.01	0.734		0.03	0.438		0.07	0.260		0.03	0.417		0.11	0.137		0.07	0.230	

Significant values p≤0.05 are in bold, marginally significant values p≤0.1 are in italics. Df Error = 19. Dir. indicates direction of the relationship for significant results, where+means that larger plants perform better and – indicates that smaller plants perform better in the given seed rain intensity treatment compared to medium seed rain intensity treatment. Subs. indicates nutrient-poor and nutrient-rich substrates.

## Discussion

This study demonstrates that seed rain intensity has a significant effect on species richness (in early stages of community development) and primarily on the composition of plant communities. The effect on species composition in fact even gets stronger over time and is the strongest in times 3, 5 and 6. I thus assume that while the effects of species richness are very transient, the effects on species composition may be persisting for extended time period. These findings provide a novel possible explanation for differences in species richness and mainly in species composition in the absence of differences in environmental conditions. Specifically, it suggests that differences in the intensity of seed rain, which are likely to occur (e.g., at localities with different degrees of isolation from seed sources), may lead to very different plant communities even when the habitat conditions are exactly the same cf. [7].

In this study, I mimicked different intensity of seed rain into a newly created habitat. In reality, differences in seed rain intensity may be observed at habitats with different isolation. Strong effect of habitat isolation on species composition was detected in the study region during our previous studies [53], [50] as well as in other systems, e.g. [56], [57]. It has been assumed that the ability to disperse is the key trait explaining differences in species composition in relation to isolation, e.g. [58], [59]. However, here I demonstrate that the overall intensity of the seed rain and the resulting intensity of competition may explain these differences in species composition. Thus, the key trait explaining a species' response to isolation may not be only the ability to reach the isolated habitat but also plant size, which likely corresponds to a species' competitive ability, e.g. [37], [45]. In real situation, both the effects of seed rain intensity will, however, always be linked to effects of differential seed dispersal capacity, and separating the contribution of these two factors in a natural setting will thus be difficult.

Indeed, habitats with lower seed rain intensity hosted smaller species that likely had lower competitive ability under nutrient-rich conditions. Thus, isolated habitats may serve as refuges for weak competitors under the condition they are also good dispersers. A similar mechanism for survival of species with low competitive ability was described as the competition–colonization trade-off, e.g. [21], [22]. Surprisingly, there was, however, an opposite trend in the nutrient-poor soil. Here, larger species were prevailing in low seed rain intensity treatment. A possible explanation if this pattern is related to the soil used in the experiment, which is very nutrient poor and has very low water permeability. It thus represents a very extreme environment for the species. The prevalence of small species in the medium density treatment may be linked to the fact that these smaller species are only able to successfully establish if the extreme conditions are meliorated by the presence denser vegetation cover, so that the larger species facilitate the establishment of the small ones. This can happen because the larger species likely grow faster and have higher change to quickly overcome the very sensitive seedling stage. In the nutrient poor substrate, larger species also prevail in the highest density treatment. Here, the pattern thus corresponds to the expected pattern under competitive interactions and to the pattern found in the nutrient-rich soil when comparing low and medium density.

In spite of the strong effect of seed rain intensity on species identity, its effect on species richness was relatively weak. The results of the experiment on the nutrient-rich substrate do not support the prediction that due to higher competition for light on nutrient-rich soil, species richness will be the highest in the lowest seed rain intensity treatment. On nutrient-poor soil, species richness seems to be the highest at the medium seed rain intensity (even though it was not significantly different in most time periods), indicating that species richness in this treatment reflects the balance between competition for nutrients, limitations caused by the availability of propagules and their ability to establish or possible positive interactions between the species. This medium seed rain intensity treatment also hosts the smallest plants in nutrient-poor soil.

The strong differences in the responses of richness and species identities to seed rain intensity in the two nutrient treatments indicates that nutrient availability greatly modifies the effects of seed rain intensity. Similarly, Brewer [60] demonstrated that other species will profit from a disturbance (modifying the intensity of competition in a way similar to seed rain intensity) under different nutrient regimes.

The effects of seed rain intensity are thought to be mediated via differences in biomass [38], [61], [47], [62]. In our study, seed rain intensity had a significant effect on aboveground biomass only in the first six months. Later, the difference in biomass between the different seed rain intensity treatments was counterbalanced by higher individual mortality and slower growth in the high seed rain intensity treatment. These initial differences in aboveground biomass could theoretically explain the effects of seed rain intensity on species richness. However, the effect of seed rain intensity on species richness remained almost unchanged after using aboveground biomass as a covariate in our models, suggesting that the effect of seed rain intensity on species richness is independent of aboveground biomass. However, the absence of differences in aboveground biomass does not exclude differences in belowground biomass, and thus, intensive belowground competition is a possible factor explaining the effects of seed rain intensity. The effect of seed rain intensity on species richness may also involve other factors such as differential germination and growth rates, differences in positive interactions among species, intensity of resource use [63], [61], secretion of allelochemicals by various species [64] or differential attraction of herbivores [65].

The relatively weak effects of seed rain intensity on number of species, in combination with strong effects on traits of the species, indicate that seed rain intensity leads to species exchange rather than to the elimination of some species. This is supported by the significant effect of seed rain intensity on species composition throughout the experiment. The effect of seed rain intensity on species composition was of the same magnitude as the effect of substrate (in the 1^st^ time period it was even stronger). This suggests that the effect of seed rain intensity may be as important for the composition of natural communities as the commonly considered effect of substrate, e.g. [66], [67], [68]. Interaction between effects of seed rain interacts and effect of habitat conditions to dictate species composition was also demonstrated by Foster et al. [69].

In this experiment, I was adding seeds into bare soil and thus follow development of communities from the beginning. In contrast, the seed addition experiments usually add seeds to already existing communities and thus follow the interactions between the existing vegetation and the newly arriving species (reviewed by Myers & Harms [3]). The effects detected in the study are thus effects under lower competition than when the seeds are added into established vegetation. However, the densities used in this study lead to fast development of aboveground biomass, high size differentiation among the plants and high competition already at the end of the first field season and thus the processes in the experimental communities are likely quite similar to conditions in the field experiments. Of course the established vegetation will likely limit the seedlings also by resource depletion. Such effect of vegetation, can be, however, viewed as effect of change in carrying capacity of the whole system. The differences in the effects of seed rain intensity between our experimental community and established vegetation are thus likely similar to the differences between the two soil types used in our study.

A theoretical explanation for the relationship between sowing density and species richness could also be sampling effect, i.e. limitation by number of individuals present in each experimental community, e.g. [70], [71]. This explanation is, however, not likely, since number of individuals in all communities was always more than a hundred (the experimental communities are relatively large compared to plan size). In addition, the effects on species richness were only transient.

## Conclusions

The results of this study provide novel insights into the importance of seed rain intensity for community composition. The critical importance of seed rain intensity for the composition of the resulting community clearly demonstrates that future seed addition experiments need to carefully consider the amount of seed added to reflect the amount that can be naturally achieved in the field. The intensity of the seed rain is also a possible candidate for explaining differences in the composition of natural communities in the absence of environmental differences. Furthermore, these results have important possible implications for studies of the consequences of habitat fragmentation for natural communities. Specifically, they suggest that by increasing fragmentation, and consequently lowering the intensity of the seed rain, habitat fragmentation may lead to communities with different species compositions that cannot be explained by differences in the dispersal abilities of these species.

## Supporting Information

Figure S1 **Effect of seed rain intensity, substrate and time on A) aboveground biomass and B) species richness.** This figure is based on the same data as Fig. 2 but is sorted by time and by not seed rain intensity.
(DOC)

Figure S2 **Effect of seed rain intensity on frequency of recordings of selected species in different seed rain intensity treatments summed over all time periods and substrates.**
(DOC)

Figure S3 **Effect of seed rain intensity on species composition of the experimental communities determined using canonical correspondence analysis (CCA) based on presence/absence data over all the time periods.**
(DOC)

Table S1 **List of species used in the study.**
(DOC)

Table S2 **Comparison of soil properties in the nutrient-rich and nutrient-poor treatment.**
(DOC)

Table S3 **Effect of seed rain intensity and substrate on species richness.** Total biomass in the pot in the given time period was used as a covariate in all the tests.
(DOC)
