# Supplementary material for: Seed Density Significantly Affects Species Richness and Composition in Experimental Plant Communities
Source: PLoS One. 2012 Oct 15;7(10):e46704. doi: 10.1371/journal.pone.0046704 (PMC3471906; doi:10.1371/journal.pone.0046704)
Supplement: Figure S1 — Effect of seed rain intensity, substrate and time on A) aboveground biomass and B) species richness. This figure is based on the same data as Fig. 2 but is sorted by time and by not seed rain intensity. (DOC) [file pone.0046704.s001.doc]

Figure S1. Effect of seed rain intensity, substrate and time on A) aboveground biomass and B) species richness. The graphs show mean with 95% confidence intervals. This figure is based on the same data as Fig. 2 but is sorted by time and not by seed rain intensity. Low, medium and high indicates low, medium and high seed rain intensity treatment.

A)

B)
