# Supplementary material for: Seed Density Significantly Affects Species Richness and Composition in Experimental Plant Communities
Source: PLoS One. 2012 Oct 15;7(10):e46704. doi: 10.1371/journal.pone.0046704 (PMC3471906; doi:10.1371/journal.pone.0046704)
Supplement: Figure S2 — Effect of seed rain intensity on frequency of recordings of selected species in different seed rain intensity treatments summed over all time periods and substrates. (DOC) [file pone.0046704.s002.doc]

Figure S2. Effect of seed rain intensity on frequency of recordings of selected species in different seed rain intensity treatments summed over all time periods and substrates. Eleven species with the strongest response to seed rain intensity are plotted. Differences in number of recordings within each species were tested using χ2 test. Significant values (p ≤ 0.05) are marked with *, marginally significant values (p ≤ 0.1) are marked with +. High, medium and low corresponds to high, medium and low seed rain intensity treatment. The abbreviations indicate species names: Asp cyn - *Asperula cynanchica*, Cam glo - *Campanula glomerata*, Car tom - *Carex tomentosa*, Cir pan - *Cirsium pannonicum*, Cor var - *Coronilla varia*, Pri ver - *Primula veris*, Sal pra - *Salvia pratensis*, Sal ver - *Salvia verticilata*, Sta rec - *Stachys recta*, Teu cha - *Teucrium chamaedris* and Tri mon - *Trifolium montanum*.
