# Supplementary material for: Seed Density Significantly Affects Species Richness and Composition in Experimental Plant Communities
Source: PLoS One. 2012 Oct 15;7(10):e46704. doi: 10.1371/journal.pone.0046704 (PMC3471906; doi:10.1371/journal.pone.0046704)
Supplement: Figure S3 — Effect of seed rain intensity on species composition of the experimental communities determined using canonical correspondence analysis (CCA) based on presence/absence data over all the time periods. (DOC) [file pone.0046704.s003.doc]

Figure S3. Effect of seed rain intensity on species composition of the experimental communities determined using canonical correspondence analysis (CCA) based on presence/absence data over all the time periods. The 1^st^ axis explains 2.2% and the 2^nd^ 0.6% of the total variation in the dataset. The variation explained corresponds to 10.84% and 2.96% of variation which could be explained by two ordination axes in an indirect (DCA) analysis. High, medium and low corresponds to high, medium and low seed rain intensity treatment. The abbreviations indicate species names: Asp cyn - *Asperula cynanchica*, Ast cic - *Astragalus cicer*, Bup fal - *Bupleurum falcatum*, Cam glo - *Campanula glomerata*, Car tom - *Carex tomentosa*, Cir pan - *Cirsium pannonicum*, Cen jac - *Centaurea jaceae*, Cen sca - *Centaurea scabiosa*, Cor var - *Coronilla varia*, Inu hir - *Inula hirta*, Med fal - *Medicago falcata*, Pri ver - *Primula veris*, Sal pra - *Salvia pratensis*, Sal ver - *Salvia verticilata*, Sta rec - *Stachys recta*, Tan cor - *Tanacetum corymbosum*, Teu cha - *Teucrium chamaedris*, Thy pul - *Thymus pulegioides* and Tri mon - *Trifolium montanum*. Position of the triangles indicates relationship of the species to the seed rain intensity treatments.
