# Supplementary material for: Seed Density Significantly Affects Species Richness and Composition in Experimental Plant Communities
Source: PLoS One. 2012 Oct 15;7(10):e46704. doi: 10.1371/journal.pone.0046704 (PMC3471906; doi:10.1371/journal.pone.0046704)
Supplement: Table S1 — List of species used in the study. (DOC) [file pone.0046704.s004.doc]

Table S1. List of species used in the study. The nomenclature follows Tutin et al. (1964-1983).

| Species | Family |
| --- | --- |
| Agrimonia eupatorium | Rosaceae |
| Anthericum ramosum | Liliaceae |
| Anthylis vulneraria | Fabaceae |
| Asperula cynanchica | Rubiaceae |
| Asperula tinctoria | Rubiaceae |
| Aster amellus | Asteraceae |
| Astragalus cicer | Fabaceae |
| Astragalus glycyphylos | Fabaceae |
| Brachypodium pinnatum | Poaceae |
| Bromus erectus | Poaceae |
| Bupleurum falcatum | Apiaceae |
| Campanula gentilis | Campanulaceae |
| Campanula glomerata | Campanulaceae |
| Centaurea jacea | Asteraceae |
| Centaurea scabiosa | Asteraceae |
| Cirsium acaule | Asteraceae |
| Cirsium pannonicum | Asteraceae |
| Coronilla varia | Fabaceae |
| Carex flaca | Cyperaceae |
| Carex tomentosa | Cyperaceae |
| Dianthus carthusianorum | Caryophyllaceae |
| Helianthemum grandiflorum | Cystaceae |
| Inula hirta | Asteraceae |
| Inula salicina | Asteraceae |
| Laserpicium latifolium | Apiaceae |
| Leontodon hispidus | Asteraceae |
| Linum flavum | Linaceae |
| Linum tenuifolium | Linaceae |
| Lotus corniculatus | Fabaceae |
| Medicago falcata | Fabaceae |
| Plantago media | Plantaginaceae |
| Primula veris | Primulaceae |
| Prunela grandiflora | Lamiaceae |
| Salvia pratensis | Lamiaceae |
| Salvia verticilata | Lamiaceae |
| Sanquisorba minor | Rosaceae |
| Scabiosa ochroleuca | Dipsaceceae |
| Stachys recta | Lamiaceae |
| Tanacetum corymbosum | Asteraceae |
| Teucrium chamaedris | Lamiaceae |
| Thymus pulegioides | Lamiaceae |
| Trifolium medium | Fabaceae |
| Trifolium montanum | Fabaceae |
| Veronica teucrium | Scrophulariaceae |
