# Supplementary material for: Seed Density Significantly Affects Species Richness and Composition in Experimental Plant Communities
Source: PLoS One. 2012 Oct 15;7(10):e46704. doi: 10.1371/journal.pone.0046704 (PMC3471906; doi:10.1371/journal.pone.0046704)
Supplement: Table S2 — Comparison of soil properties in the nutrient-rich and nutrient-poor treatment. (DOC) [file pone.0046704.s005.doc]

Table S2. Comparison of soil properties in the nutrient-rich and nutrient-poor treatment. The values are mean ± SD. N = 18. All the values are significantly different between nutrient rich and poor substrate at p ≤ 0.05. Larger value in each pair is in bold.

|  | Rich | Poor |
| --- | --- | --- |
| pH (H2O) | 7.39±0.11 | **7.93±0.13** |
| pH (KCl) | 7.14±0.06 | **7.77±0.06** |
| N (%) | **0.42±0.09** | 0.07±0.01 |
| C total (%) | **7±1.28** | 2.71±0.16 |
| C carbonate (%) | 0±0 | **2.08±0.1** |
| C organic (%) | **7±1.28** | 0.63±0.2 |
| Ca (mg/1000g) | 1862±320.35 | **7241±1685** |
| Mg (mg/1000g) | **270±4.66** | 171±14 |
| K (mg/1000g) | **308±155** | 68±7 |
| P (mg/1000g) | **102±18** | 7±2 |
