# Supplementary material for: Seed Density Significantly Affects Species Richness and Composition in Experimental Plant Communities
Source: PLoS One. 2012 Oct 15;7(10):e46704. doi: 10.1371/journal.pone.0046704 (PMC3471906; doi:10.1371/journal.pone.0046704)
Supplement: Table S3 — Effect of seed rain intensity and substrate on species richness. Total biomass in the pot in the given time period was used as a covariate in all the tests. (DOC) [file pone.0046704.s006.doc]

Table S3. Effect of seed rain intensity and substrate on species richness assessed using ANOVA. Total biomass in the pot in the given time period was used as a covariate in all the tests. The tests are done for all the time periods together (using repeated measures analyses) and for each time period separately. N = 60 for each time period. Significant values p ≤ 0.05 are in bold, marginally significant values p ≤ 0.1 are in italics, n.s. indicates p > 0.1.

|  | All | | Time 1 | | Time 2 | | Time 3 | | Time 4 | | Time 5 | | Time 6 | |
| --- | --- | --- | --- | --- | --- | --- | --- | --- | --- | --- | --- | --- | --- | --- |
|  | R^2^ | p | R^2^ | p | R^2^ | p | R^2^ | p | R^2^ | p | R^2^ | p | R^2^ | p |
| Density | 0.01 | 0.153 | **0.01** | 0.063 | **0.10** | **0.040** | **0.02** | **0.042** | *0.04* | *0.013* | 0.01 | 0.603 | <0.01 | 0.852 |
| Substrate | **0.08** | **<0.001** | **0.09** | **<0.001** | <0.01 | 0.994 | **0.61** | **<0.001** | **0.37** | **<0.001** | **0.57** | **<0.001** | **0.51** | **<0.001** |
| Density × substrate | <0.01 | 0.469 | <0.01 | 0.374 | 0.05 | 0.178 | **0.03** | **0.004** | 0.01 | 0.540 | 0.01 | 0.388 | **0.12** | **<0.001** |
